# Genome-Wide Identification of the Defensin Gene Family in *Triticum durum* and Assessment of Its Response to Environmental Stresses

**DOI:** 10.3390/biology14040404

**Published:** 2025-04-11

**Authors:** Nawress Gamas, Fahmi Smaoui, Walid Ben Romdhane, Alina Wiszniewska, Narjes Baazaoui, Mohamed Taieb Bouteraa, Yosra Chouaibi, Anis Ben Hsouna, Miroslava Kačániová, Maciej Ireneusz Kluz, Stefania Garzoli, Rania Ben Saad

**Affiliations:** 1 Biotechnology and Plant Improvement Laboratory, Centre of Biotechnology of Sfax, University of Sfax, B.P “1177”, Sfax 3018, Tunisia; gamasnawres1408@gmail.com (N.G.); bouteraa.taieb@gmail.com (M.T.B.); yosrachouaibi1161@gmail.com (Y.C.); benhsounanis@gmail.com (A.B.H.); raniabensaad@gmail.com (R.B.S.); 2 Faculty of Sciences of Gafsa, University of Gafsa, Sidi Ahmed Zarrouk, Gafsa 2112, Tunisia; 3 Research Laboratory “Microorganisms and Human Disease LR03SP03”, Laboratory of Microbiology, Habib Bourguiba University Hospital, University of Sfax, Sfax 3029, Tunisia; smaouifahmi@yahoo.fr; 4 Department of Plant Production, College of Food and Agricultural Sciences, King Saud University, P.O. Box 2460, Riyadh 11451, Saudi Arabia; walid.brm3@gmail.com; 5 Department of Botany, Physiology and Plant Protection, University of Agriculture in Kraków, 31-120 Kraków, Poland; alina.wiszniewska@urk.edu.pl; 6 Biology Department, Faculty of Science, King Khalid University, Abha 61421, Saudi Arabia; nhrmbaazoui@kku.edu.sa; 7 Faculty of Sciences of Bizerte UR13ES47, University of Carthage, BP W, Bizerte 7021, Tunisia; 8 Department of Environmental Sciences and Nutrition, Higher Institute of Applied Sciences and Technology of Mahdia, University of Monastir, Mahdia 5100, Tunisia; 9 Institute of Horticulture, Faculty of Horticulture and Landscape Engineering, Slovak University of Agriculture, Tr. A. Hlinku 2, 94976 Nitra, Slovakia; 10 School of Medical and Health Sciences, University of Economics and Human Sciences in Warsaw, Okopowa 59, 01-043 Warszawa, Poland; 11 Andrzej Frycz Modrzewski Krakow University, Gustawa Herlinga-Grudzińskiego 1, 30-705 Kraków, Poland; 12 Department of Chemistry and Technologies of Drug, Sapienza University, 00185 Rome, Italy; stefania.garzoli@uniroma1.it

**Keywords:** *Triticum turgidum* ssp. *durum*, *TdPDF* genes, genome-wide analysis, host–pathogen interaction, abiotic stress

## Abstract

Plant defensins (PDFs) are a group of cationic antimicrobial peptides that are distinguished by their unique tertiary structure and play significant roles in physiological metabolism, growth, and stress tolerance. Defensins are key components of plant innate immunity; they can target a wide variety of microorganisms. This study aimed to identify and investigate the role of *Triticum durum* PDFs (*TdPDFs*) in response to environmental stresses. Prior to this, in silico analyses of *TdPDF* genes were conducted to assess their chromosomal locations, conserved motifs, exon–intron distribution, and cis-regulatory elements in the promoter regions. Additionally, bioinformatic analyses were performed to characterize the structure of TdPDF proteins, evaluate their phylogenetic relationships, predict their subcellular localization, and estimate their physicochemical properties. Docking studies were conducted to assess the interactions between TdPDF proteins and the fungal plasma membrane. A total of 28 *TdPDF* genes were identified in durum wheat based on their conserved domain PF00304 (gamma-thionin). These genes are distributed across all chromosomes of the durum wheat genome, except for chromosomes 4A and 7A. Analysis of the promoters of these genes revealed numerous elements associated with development, hormone responsiveness, and environmental stress. The majority of TdPDF proteins were predicted to be located extracellular. In addition, TdPDF proteins were classified into three clusters based on sequence similarity. Phylogenetic analysis suggested that TdPDF proteins share ancestral similarities with the PDF sequences of other monocotyledonous species. Molecular docking studies revealed that TdPDF proteins interact with fungal plasma membranes, suggesting that they play a critical role in the resistance of plants to pathogen infections. Expression analysis underlined the crucial role of nine *TdPDF* genes in the defense responses of durum wheat against both pathogenic and environmental stressors. Overall, our findings underscore the potential of *TdPDF* genes in host-plant resistance and highlight opportunities for their application in crop improvement toward stress tolerance.

## Simple Summary

Durum wheat is an essential crop, particularly in regions with harsh climates, yet it faces threats from pathogens and environmental stressors that can reduce yield and quality. Understanding the genetic basis of stress resistance is a critical for developing more resilient varieties. Among defense-related genes, defensins play a crucial role in plant immunity by targeting fungal pathogens and enhancing stress tolerance. However, their role in durum wheat remains largely unexplored. In this study, we identified 28 defensin-related genes and analyzed their structure, evolutionary relationships, and predicted functions. These genes contain regulatory elements linked to stress responses and hormone signaling, suggesting their involvement in plant protection. Most defensin proteins were predicted to be secreted outside plant cells, where they may interact with pathogens. Computer-based modeling suggested that these proteins can bind to fungal membranes, which highlights their potential antimicrobial role in durum wheat. Further experimental analysis revealed that multiple defensin genes are highly expressed under stress conditions, underscoring their importance in protecting durum wheat from diseases and environmental challenges. These findings provide new insights into the natural defense mechanisms of durum wheat and open possibilities for developing stress-resistant varieties through breeding or biotechnology.

## 1. Introduction

Durum wheat (*Triticum durum*) is an allotetraploid species within the monocotyledonous *Triticeae* tribe, originating from the hybridization and subsequent chromosomal doubling of *Triticum urartu* and *Aegilops speltoides Tausch* [1].

Durum wheat (*Triticum turgidum* L. spp. *durum*) ranks among the world’s most widely cultivated crops [2]. However, in the Mediterranean region, its productivity is significantly constrained by environmental factors, particularly drought and salinity [3], as well as biotic stress from pathogenic fungi such as *Fusarium oxysporum* [4,5]. Under biotic stress triggered by fungi, bacteria, or viruses, plants initiate a complex molecular dialogue involving reactive oxygen species (ROS), salicylic acid (SA), jasmonic acid (JA), ethylene (ET), and nitric oxide [6]. These signaling molecules orchestrate defense responses, with SA primarily activated in response to biotrophic pathogen attacks [7] and ET modulating responses to necrotrophic pathogens and abiotic stresses [8]. The JA pathway is particularly active during herbivorous insect attacks [9,10]. The cross-talk between SA, JA, and ET synergistically enhances defensin gene expression and boosts plant immunity [7]. Plant defensins (PDFs), a key class of cysteine-rich antimicrobial peptides (AMPs), play a central role in innate plant defense [11]. Structurally, defensins adopt a conserved βαββ “knottin fold” stabilized by disulfide bonds, comprising one α-helix and three antiparallel β-sheets [12,13,14]. This structural arrangement provides the stability necessary for their effective functioning [14]. PDFs are predominantly located in the cell wall and extracellular space [15], which allows them to participate in defense responses against pathogens and pests [16]. Most plant PDFs have a biologically conserved domain known as the gamma-thonin domain (PF00304) [17]. PDF proteins serve numerous biological functions, including roles in plant development and seed growth [18,19,20]. They exhibit antifungal [21] and antibacterial activities [22], contribute to resistance against zinc [23], act as α-amylase inhibitors [24], and possess antioxidant properties with low cytotoxic effects [25]. In addition, several studies have shown that PDF proteins are responsible for stress tolerance to salinity, drought, and cold [23,26,27]. The first *PDF* gene was identified in seeds of monocot and dicot species [15]. Their diversification has evolved in response to environmental challenges and microbial threats [28]. The protein from chickpeas induces resistance against *F. oxysporum f.* sp. *ciceris* and *R. bataticola* [29]. The ZmD32 defensin protein showed a similar response against *Candida albicans*, *C. auris*, *C. glabrata*, *C. krusei*, *C. parapsilosis*, *C. tropicalis*, and *F. graminearum* [30]. Both γ1-zeathionins and γ2-zeathionins (PDC-1) act as sodium channel blockers [31,32]. In addition, the *BcDef1*, isolated from *Brugmansia candida* (*Bc*), exhibited antioxidant activity [25]. Several studies have illustrated that the constitutive expression of a radish defensin improved tomato resistance to *Alternaria solani* [33]. Moreover, certain PDFs demonstrate crucial activities, such as protease inhibitors [34] and anti-cancer agents [35].

Although PDFs have been studied in other species, their presence and function in durum wheat remain largely unexplored. In this context, advances in genomic technologies and databases offer new opportunities to identify novel genes, investigate their regulatory mechanisms, and explore potential interactions with other stress-response genes. This study aimed to leverage genomic data to identify and characterize *PDF* genes and proteins in durum wheat, including their chromosomal localization, conserved motifs, cis-regulatory elements, phylogenetic relationships, structural properties, and protein interactions. Additionally, it explored their potential roles and regulatory mechanisms under various biotic and abiotic stress conditions through bioinformatics analyses and qPCR-based expression profiling. This comprehensive analysis of TdPDFs across the *T. durum* genome aimed to enhance our understanding of plant disease and stress-resistance genes, ultimately supporting the development of improved wheat varieties.

## 2. Materials and Methods

### 2.1. Plant Material and Stress Treatments

The experiment was carried out using the widely cultivated Tunisian durum wheat variety ‘Karim’ (*Triticum turgidum* L. subsp. *durum* (*Desf.*) *Husn.*), with seeds sourced from Centre d’AppuiChebika-CRDA Kairouan, Tunisia. The seeds were sterilized in 70% ethanol for 1–2 min, followed by rinsing with sterile water, and then allowed to germinate in Petri dishes for 7 days to produce seedlings [36]. To examine the response of 9 *TdPDF* genes to stress (Table S1), ten-day-old seedlings grown hydroponically in a nutrient solution were subjected to different stress conditions, including salinity, osmotic, cold, abscisic acid (ABA), salicylic acid (SA), and methyl jasmonate (MeJA). Salt (150 mM NaCl) and osmotic stress (20% (*w*/*v*) PEG 6000) were applied as described by Ben Romdhane et al. [37]. For cold treatment, the seedlings were exposed to 4 °C. The plants were maintained in a controlled environment chamber (phytotron) with the following settings: temperature (25 ± 5 °C), light intensity (280 µmol m^−2^ s^−1^), photoperiod (16 h light/8 h dark), and relative humidity (60 ± 10%). The leaves were sprayed with the phytohormones ABA (100 µM), SA (100 µM), and MeJA (100 µM), while a control group received a water spray.

The *F. graminearum* strain was grown on potato dextrose agar (PDA) plates at 30 °C for seven days. Afterward, fifteen-day-old plants were inoculated with a freshly prepared spore suspension (2 × 10^7^ spores/mL) in Hoagland’s solution. Following inoculation, plants were kept in high humidity at 25 °C under a 16 h photoperiod. Harvests were made at 1, 3, 24, and 48 h post-inoculation to evaluate infection development and the plant’s defense responses. To analyze tissue-specific gene expression, samples were collected from various plant parts (leaves, stems, roots, spikes, anthers, and developing seeds at 21 days post-anthesis) from greenhouse-grown plants. Each tissue was collected separately, frozen in liquid nitrogen immediately, and stored at −80 °C to preserve RNA integrity.

### 2.2. Identification and Chromosomal Mapping of TdPDF Genes in Durum Wheat

The TdPDF protein sequences were retrieved from the Ensembl Plants database (https://plants.ensembl.org/index.html; accessed on 22 January 2025), while the TaPDF protein sequences [17] were sourced from the Grain Genes database (https://wheat.pw.usda.gov/GG3; accessed on 22 January 2025) and served as query sequences for searching TdPDF proteins via the BLAST tool version 1.4.0, employing an expected E-value threshold of E^−50^. Then, the full sequences of TdPDF proteins were screened using SMART (https://smart.embl-heidelberg.de/, accessed on 22 January 2025) and INTER PRO to identify the PF00304 gamma-thionin domain. The chromosomal positions of all *TdPDF* genes were plotted using the PhenoGram Plot tool (https://visualization.ritchielab.org/phenograms/plot, accessed on 22 January 2025).

### 2.3. Characterization of TdPDF Proteins

The physicochemical properties of the TdPDF proteins, including the molecular weight (MW), instability index (II), isoelectric point (pI), and grand average of the hydropathicity (GRAVY) of TdPDF proteins, were determined by the ProtParam tool within the ExPASy bioinformatics web tool (https://web.expasy.org/protparam/, accessed on 22 January 2025, [38]). In addition, to identify the subcellular localization of the TdPDF proteins, the BUSCA web tool (https://busca.biocomp.unibo.it/, accessed on 22 January 2025) was employed using the “Eukarya—plant—16 compartments” taxonomic origin option [39]. The signal peptide of TdPDF was predicted using SignalP5.1 (https://www.cbs.dtu.dk/services/SignalP/, accessed on 22 January 2025, [40]) by selecting “Eukarya” as the organism group.

### 2.4. Phylogenetic Analysis

The protein sequences of PDFs from *Arabidopsis thaliana* (TAIR database) (https://www.Arabidopsis.org/index.jsp, accessed on 22 January 2025), *Triticum aestivum*, and *Oryza sativa* were retrieved from Ensembl Plants. Multiple alignments of the PDF sequences were performed using the Clustal W algorithm for protein sequences using MEGA11 [41]. Maximum likelihood phylogenetic analysis of the TdPDF sequences was conducted using 1000 bootstrap replicates in MEGA11, incorporating the PDF sequences of *Arabidopsis thaliana*, *Oryza sativa*, and *Triticum aestivum* as outgroups.

The resulting protein trees were visualized using the Interactive Tree of Life tool (iTOL, available at https://itol.embl.de/, accessed on 22 January 2025, [42]). Additionally, the conservation of the TdPDF motif was assessed using the MEME v5.4.1 tool and visualized alongside the trees (https://meme-suite.org, accessed on 22 January 2025, [43]).

### 2.5. Analyses of TdPDF Genes and Promoter Regions

The Plant Compara tool from the Ensembl Plants database was employed to identify homologs, paralogs, and orthologs for each gene. Additionally, duplication events impacting these genes were investigated using TBtools v1.095 software [44] to analyze the associated evolutionary pressures. To examine the exon–intron structures of *TdPDF* genes, the Gene Structure Display Server (GSDS) 2.0 (https://gsds.gao-lab.org, accessed on 22 January 2025) was employed [45].

To analyze the putative cis-acting regulatory elements in the promoter regions, the 2 kb sequences upstream of each gene, obtained from the Ensembl Plants database, were examined using the PlantCARE (https://bioinformatics.psb.ugent.be/webtools/plantcare/html, accessed on 22 January 2025) and New PLACE (https://www.dna.affrc.go.jp/PLACE/?action=newplace, accessed on 22 January 2025) databases.

### 2.6. Quantitative RT PCR Analyses

Total RNA was extracted from plant tissues using TRIzol reagent (Invitrogen, Carlsbad, CA, USA), followed by treatment with DNase I (MBI Fermentas, Hanover, MD, USA) at 37 °C for 15 min to eliminate residual genomic DNA. Two micrograms of RNA were reverse-transcribed into cDNA using M-MLV reverse transcriptase (Thermo Fisher Scientific, Waltham, MA, USA). The resulting cDNA was diluted 1:5 and subjected to amplification with gene-specific primers designed using Primer3 (https://primer3plus.com/cgi-bin/dev/primer3plus.cgi, accessed on 22 January 2025) and SYBR Green RT-PCR master mix (Roche Diagnostics, Mannheim, Germany). Quantitative RT-qPCR was performed using the LightCycler 480 system (Roche Diagnostics International Ltd., Rotkreuz, Switzerland) in triplicate, following the protocol described by Ben Saad et al. [46]. The thermal cycling conditions included an initial denaturation at 95 °C for 3 min, followed by 40 cycles of 95 °C for 20 s, 60 °C for 30 s, and 72 °C for 1 min. To confirm primer specificity, a melting curve analysis was conducted after the 40 cycles.

The relative expression levels of the nine *TdPDF* genes were quantified using the 2^−ΔΔCT^ method, where CT denotes the cycle threshold, as outlined by Livak and Schmittgen [47]. Expression levels were normalized to the *CDC* gene (Ta54227), a cell division control protein from the AAA-superfamily of ATPases, as reported by Giménez et al. [48]. Results represent the mean relative expression ratios obtained from three independent experiments, each with three biological replicates.

### 2.7. Docking Studies and Structural Modeling

The SWISS-MODEL server (https://swissmodel.expasy.org/interactive, accessed on 22 January 2025, [49]) was employed to predict the three-dimensional structures of TdPDFs. The full protein was modeled using Alpha Fold [50] generated templates due to the lack of experimental structures. Structures with additional domains compared to the other models were characterized using the pfam server [51]. The defensin domain was modeled using the crystal structures of the rice defensin OsAFP1 (PDB ID: 6lcq) and grapevine defensin VvK1 (PDB ID: 7c31) as templates. The resulting structures were visualized with Schrödinger Maestro (Academic version 13). Defensin domain homology structures were clustered based on TM scores using the TM-align algorithm [52]. Disulfide bond predictions were conducted using the DI pro tool (available at https://scratch.proteomics.ics.uci.edu/, accessed on 22 January 2025).

Molecular docking studies were conducted on a subset of modeled TdPDF proteins, using phosphatidylinositol 4,5-bisphosphate (PIP2) as the ligand, extracted from the crystal structure of the plant defensin NaD1 in complex with PIP2 (PDB ID: 4CQK). The docking analysis was performed using Auto Dock Vina [53]. The binding site was inferred from the experimental structure of NaD1 with PIP2 (4CQK). For TdPDF9, the docking grid was centered at (9.444, 19.556, 28.139) with dimensions of 28 × 34 × 26, while for TdPDF20, the grid was centered at (9.444, 20.528, 24.472) with dimensions of 22 × 26 × 26. In both cases, an energy range of 4 and an exhaustiveness value of 8 were applied. Visualization and analysis of 2D molecular interactions were carried out using Schrödinger Maestro (Academic version 13).

### 2.8. Assessments of Protein-Protein Interaction Network 

Protein-protein interaction analysis of TdPDF proteins was performed using the STRING database (https://string-db.org/, accessed on 22 January 2025, [54]). The interaction networks were predicted based on the closest orthologs of TdPDF proteins in *Triticum aestivum* due to the lack of *Triticum durum* data in the database. The analysis was performed using the full STRING network type with a medium confidence threshold of 0.400 and a maximum of 10 interactions to identify and visualize both direct and indirect protein associations.

## 3. Results

### 3.1. Screening and Identification of TdPDF Genes in Durum Wheat

**Figure 1:**
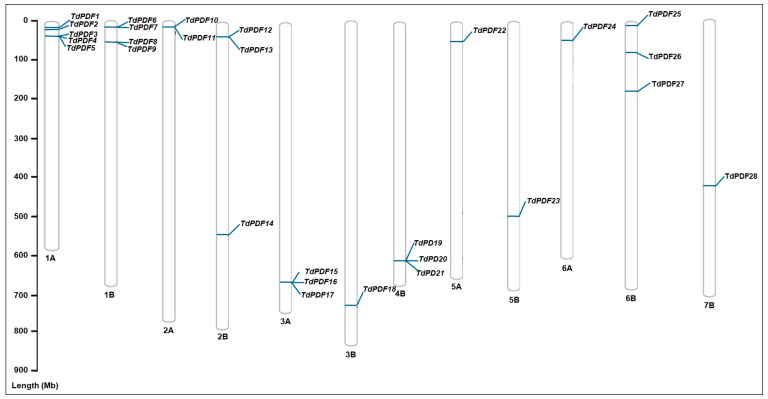
Locations of the 28 *TdPDF* genes on durum wheat chromosomes. The scale on the left represents the length of the chromosomes. Mb = million base pair.

In the present study, we identified 28 PDF genes containing the conserved domain PF00304 (gamma-thionin). These genes were named according to their chromosomal locations (TdPDF1–28). TdPDF genes are distributed in all chromosomes in the durum wheat genome except for chromosomes 4A and 7A. Each chromosome harbors multiple TdPDF genes, as illustrated in Figure 1. Notably, our findings demonstrate that many TdPDF genes located on the same chromosome appear to be in close proximity to one another. Additional details regarding the positions and characteristic features of these. genes can be found in Table 1.

### 3.2. TdPDF Protein Characterization and Subcellular Localization Prediction

The in silico analysis indicated that the length of TdPDF proteins ranges from 74 (TdPDF14and TdPDF9) to 123 (TdPDF17) amino acids. Their molecular weights vary from 7.942 kDa (TdPDF9) to 13.426 kDa (TdPDF17). The isoelectric points (pI) of these proteins also show considerable variation, with values between 5.55 (TdPDF27) and 9.79 (TdPDF4). Moreover, a value of pI below 9 (19 of the total proteins analyzed) indicates that these proteins are acidic, while PDF proteins with pI above 9 (9 of the total proteins) are basic (Table 2). Regarding protein stability, 15 out of the 28 TdPDF proteins were predicted to be stable in a test tube, as their instability index was below 40. Based on the grand average of hydropathicity (GRAVY) index, 18 of the proteins were classified as hydrophobic, while 10 were identified as hydrophilic. Most TdPDF proteins were predicted to be located in the extracellular space, with the exception of TdPDF17, which was localized in the mitochondrion (Table 2).

### 3.3. Conserved Motif and Phylogenetic Analysis of TdPDF Proteins

**Figure 2:**
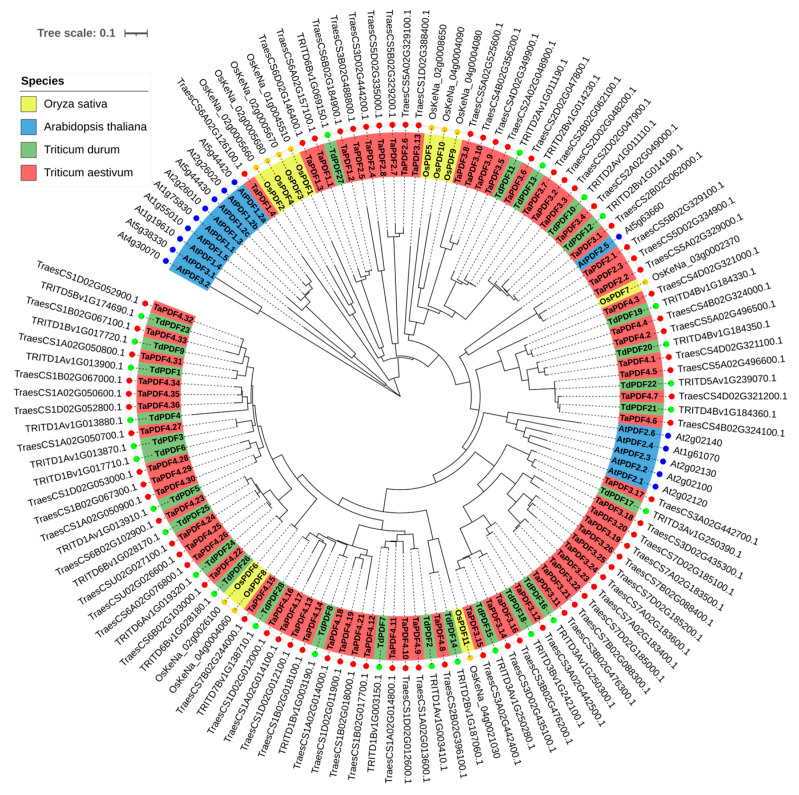
Phylogenetic tree analysis of *A. thaliana*, *T. aestivum*, *O. sativa*, and *T. turgidum* PDF proteins. The four subgroups of PDFs are presented with different colors. Red squares refer to *T. aestivum* (TaPDF) proteins, red circles for *A. thaliana* (AtPDF) proteins, red stars for *T. turgidum* (TdPDF) proteins, and red triangles for *O. sativa* (OsPDF).

To examine the evolutionary relationships between TdPDF proteins in durum wheat and other species, a phylogenetic tree was constructed with PDF protein sequences from *T. aestivum* (*n* = 73), *T. turgidum* (*n* = 28), *A. thaliana* (*n* = 15), and *O. sativa* (*n* = 11). The results suggest that TdPDF proteins share ancestral similarities with PDF sequences from other monocotyledonous species (Figure 2). The TdPDF proteins were classified into three clusters (C1, C2, and C3) based on genetic distance, and the three clusters were subdivided into six sub-clusters.

**Figure 3:**
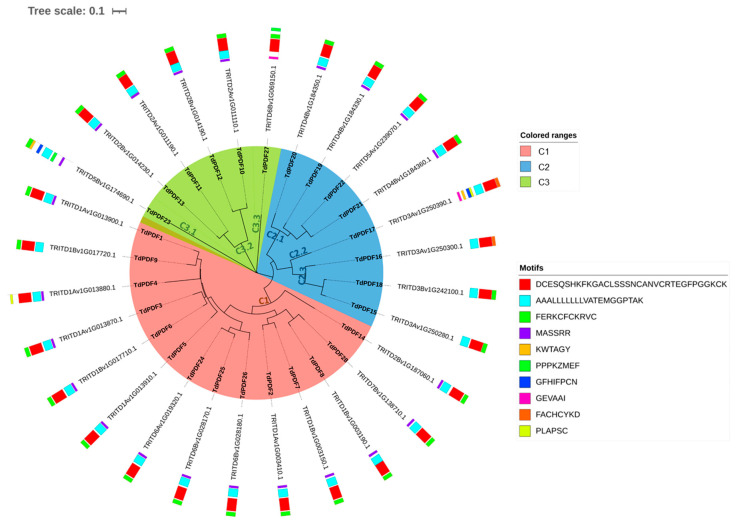
Analysis of *Triticum durum* TdPDF protein structures. The circular tree was generated using TdPDF protein sequences.

The analysis of the motif organization of TdPDF proteins revealed that they share Motif 2 (blue, Figure 3). Motif 3 was conserved across all TdPDF proteins, except for three sequences (TdPDF4, TdPDF16, and TdPDF17). In contrast, Motif 1 (red) was present in all sequences, except for one sequenceTdPDF23, while Motif 4 is conserved in 22 of the detected proteins.

### 3.4. TdPDF Gene Duplication and Ka/Ks Analysis

Gene duplication (GD) shaped the evolution of genomes in plants. This phenomenon might occur via several mechanisms, including whole genome duplication (WGD), single-gene duplications, and segmental duplications [55]. WGD serves as a significant factor in biological complexity, evolutionary innovation, and adaptation to diverse environmental conditions [56]. In T. durum, we identified eight pairs of TdPDF homologs that resulted from the whole-genome duplication (WGD) event, as well as 12 pairs of paralogs that most probably originated from segmental duplication (Table 3). The analysis of Ka/Ks ratios indicated that only the pair TdPDF7/TdPDF28 exhibited a value greater than 1 (Table 4), suggesting these genes have evolved during a period of positive selection, which accelerates evolution [57]. Conversely, all other gene pairs displayed Ka/Ks values below 1, with the highest ratios recorded at 0.7835 for TdPDF20/TdPDF21 (Table 4). These lower ratios imply that these gene pairs have evolved under strong purifying selection [57].

### 3.5. Gene Structure and Cis-Regulatory Element Analyses

**Figure 4:**
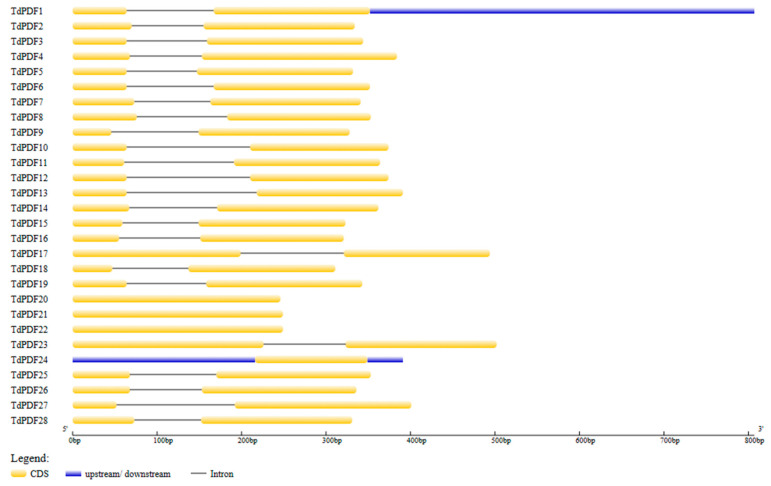
Exon–intron structure analysis of *TdPDF* genes.

The genomic information of TdPDF genes was used to map gene structure using GSDS2.0. The analysis revealed that 24 out of 28 TdPDF genes consist of two exons separated by an intron, with coding regions located at their 5′ ends (Figure 4). The remaining genes, TdPDF20, TdPDF21, TdPDF22, and TdPDF24, contain only an encoding sequence (exon). Additionally, all TdPDF genes contained the same number of exons as their Triticum aestivum orthologs, except for the orthologous pairs TdPDF21–TaPDF14, which have one and four exons, respectively, and TdPDF15–TaPDF10, which have two and three exons, respectively (Table 3).

Cis-acting regulatory elements play a critical role in the regulation of gene expression under biotic and abiotic stresses [58] and the induction of related genes by supervising promoter efficiency [59]. The analysis of sequence 2 kb upstream of TdPDF genes revealed multiple cis-elements, which were classified into three categories. Regarding the category of hormone-responsive elements, abscisic acid (ABRE, ABRE3a, ABRE4a, and ABRE2) and methyl jasmonate (MeJA, CGTCA-motifs, and TGACG-motifs) cis-elements were frequently detected across TdPDF genes. Auxin (TGA element, Aux core), salicylic acid (TCA element), gibberellic acid (Pbox, Gare motif), and ethylene (ERE) cis-elements were also detected, although less frequently.

**Figure 5:**
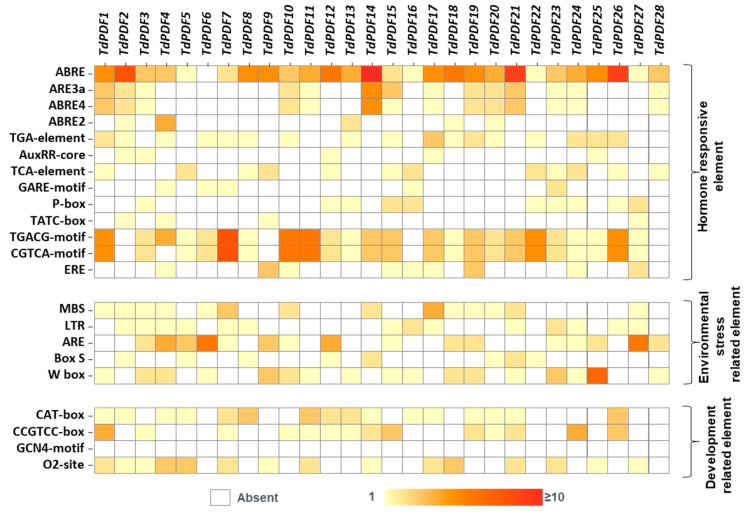
Heatmap of the cis-acting elements in the promoter regions of *TdPDF* genes.

Additionally, cis-acting regulatory elements associated with environmental stress were identified, including those related to drought (MBS), low temperature (LTR), anaerobic induction (ARE), and biotic stress (box-S, W-box). Furthermore, developmental-related cis-elements were detected, such as those involved in meristem function (CAT-box and CCGTCC-box), endosperm regulation (GCN4 motif), and metabolic regulation (o_2_-site) (Figure 5).

### 3.6. Docking Analysis and Structural Modeling

**Figure 6:**
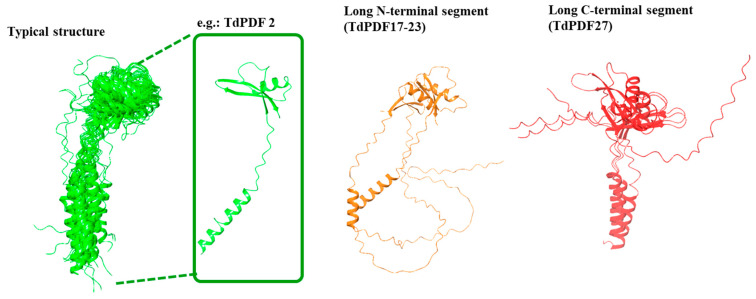
Structural characterization of the full protein models of durum wheat (*Triticum durum*) plant defensins.

The structural modeling of the full TdPDF proteins revealed that the most common conformation consisted of an α-helix representing the signal peptide, which had a relatively consistent length (around 20–35 amino acids), followed by the defensin domain, which included one α-helix and three antiparallel β-sheets (Figure 6). This conformation was observed in 25 out of 28 models, with only three deviating from this structure.

**Figure 7:**
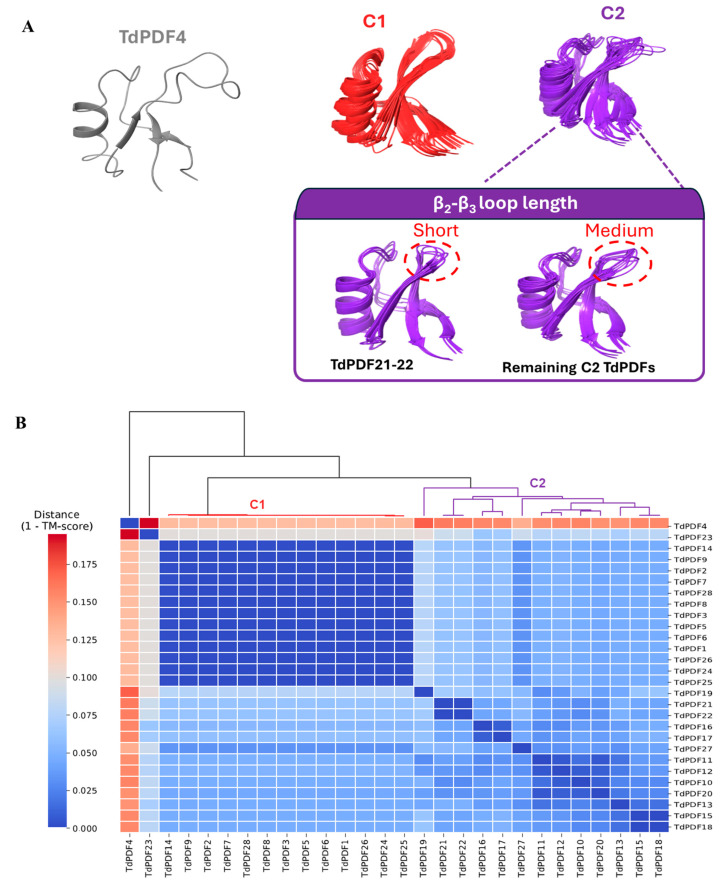
Structural clustering (**A**) and 3D representation (**B**) of the defensin domain in modeled structures of durum wheat (*Triticum durum*) plant defensin proteins.

Specifically, for two models (TdPDF17 and TdPDF23), the N-terminal α-helix segment was long, exceeding 70 amino acids. Additionally, the TdPDF27 structure featured a long segment extending toward the C-terminal region of the protein, located after the β-sheets. Regarding the defensin domains, structural clustering based on TM score identified two major clusters, C1 and C2, along with a singleton, TdPDF4 (Figure 7A).

From a structural perspective, cluster C1 exhibited high structural similarity, characterized by a protruding β2-β3 loop due to relatively long β2 and β3 sheets. TdPDF models within cluster C2 were more structurally diverse, with the β2-β3 loop length varying considerably, ranging from short in TdPDF16 and TdPDF17 to medium (Figure 7B).

**Figure 8:**
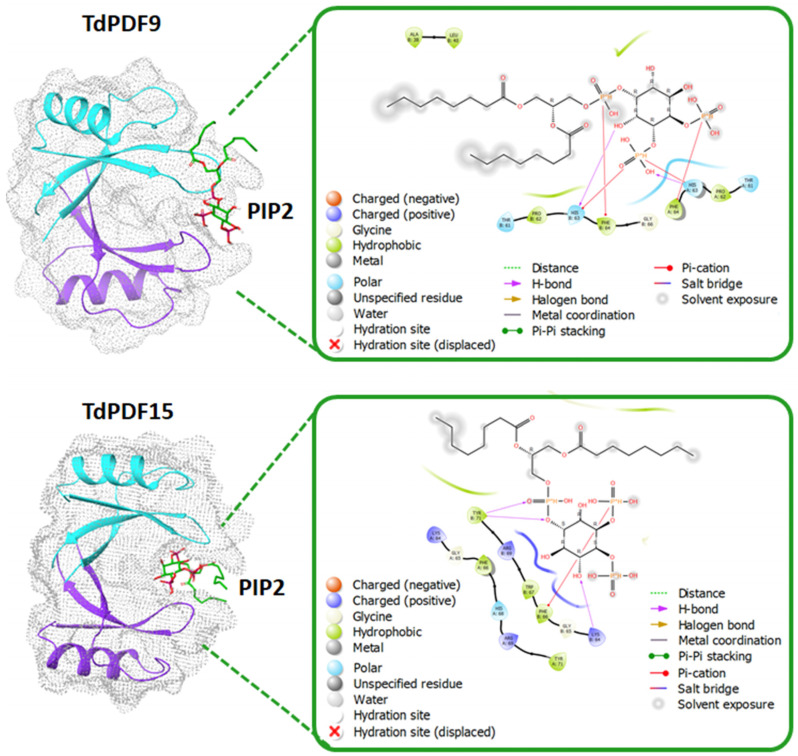
Docking analysis of TdPDF9 (representative of subcluster C2b) and TdPDF20 (representative of subcluster C2c) with phosphatidylinositol 4,5-bisphosphate (PIP2). The estimated binding affinity was −4.8 kcal/mol for TdPDF9 and −5.4 kcal/mol for TdPDF15.

Disulfide bond prediction revealed that all TdPDF structures were stabilized via two to five disulfide bonds, with a median count of four bonds. The docking analysis was performed on TdPDF9 (representative of C1) and TdPDF15 (representative of C2) with PIP2. As shown in Figure 8, the docking results suggested that PIP2 can interact with TdPDF dimers, occupying a binding pocket situated between the two monomers. The interaction between PIP2 and TdPDF is primarily mediated through hydrogen bonds and pi-cation interactions between the phosphate groups and the inositol ring of PIP2 and residues located on the β2-β3 loop of the TdPDF protein.

### 3.7. Protein-Protein Interaction Network Analysis

**Figure 9:**
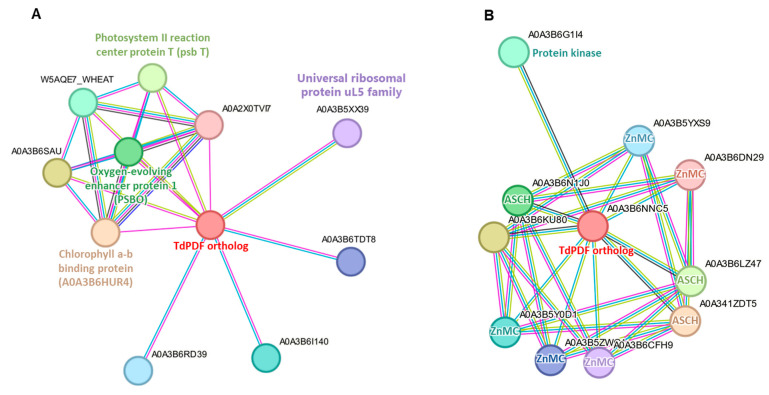
Protein-protein interaction (PPI) analysis of TdPDF protein orthologs in Triticum aestivum. (**A**) Shared protein-protein interaction network of 27 TdPDFs orthologs. (**B**) Pro-tein-protein interaction network of TdPDF27 orthologs in *Triticum aestivum*.

Protein-protein interaction analysis identified interaction networks for 28 TdPDF orthologs in Triticum aestivum. Among these, 27 TdPDFs shared a similar interaction network (Figure 9A). This network included two proteins involved in photosynthesis (photosystem II reaction center protein T and chlorophyll a–b binding protein), a protein essential for stabilizing the manganese cluster (oxygen-evolving enhancer protein 1), a member of the universal ribosomal protein uL5 family, and six uncharacterized proteins. In contrast, only TdPDF27 orthologs exhibited distinct interaction patterns (Figure 9B), engaging with three RNA-binding proteins containing the ASCH domain, five proteins possessing the ZnMc domain (zinc-dependent metalloprotease), and one protein with a kinase domain.

### 3.8. TdPDF Genes Are Highly Expressed in Developing Seeds and Induced by Multiple Stresses and Phytohormones Treatments

**Figure 10:**
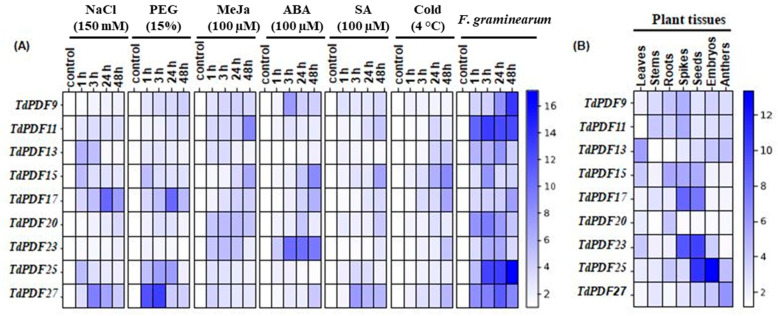
Expression pattern of durum wheat PDF genes. (**A**) Heatmap showing the expression pattern of *TdPDF* genes in *T. durum* plants subjected to 150 mM NaCl, 15% PEG−6000, 100 μM ABA, 100 μM SA, 100 µM MeJa, and *F. garminearum* for 1, 3, 24, and 48 h. (**B**) Heatmap of the expression pattern of *TdPDF* genes in roots, stems, leaves, spikes, seeds, embryos, and anthers. The specific reference CDC gene was used as an internal control for qPCR normalization. The relative expression values were calculated using the 2^−ΔΔCt^ method [47]. Three plants were used per treatment per replicate.

To investigate the functions of the TdPDF gene family in Triticum durum, we analyzed the expression levels of these genes in different tissues—leaves, stems, roots, spikes, seeds, embryos, and anthers—using RT-qPCR (Figure 10A). The results revealed that TdPDF13 showed significantly higher expression in leaves compared to the other tissues, suggesting it may be involved in leaf-specific functions such as photosynthesis, leaf development, or response to environmental factors. Moreover, TdPDF27 has the highest expression in anthers, which indicates it could control pollen development. In contrast, TdPDF25 was highly expressed in embryos, suggesting its role in early developmental processes, including embryo formation and seed maturation. TdPDF25 and TdPDF23 were highly expressed in seeds (10.78- and 10.13-fold, respectively), suggesting that these genes may play a role in seed development. TdPDF15 was predominantly expressed in roots, which might indicate its potential role in root growth and root-related processes, such as nutrient uptake or stress responses in the root system. TdPDF11 was accumulated in stems, which suggests its involvement in stem development or vascular tissue function. Overall, the differential expression of TdPDF genes across various tissues and organs points to their diverse roles in plant growth, development, and reproduction (Figure 10B). It highlights how these genes may be adapted to perform specific functions in distinct parts of the plant. For the analysis of the expression levels of nine TdPDF genes in the defense responses of durum wheat against both pathogenic and environmental stressors, we computed the average expression level among three replicates with the standard error of the mean (SEM) to help compare the expression profiles.

Next, to investigate the impact of phytohormones, as well as abiotic or biotic stress factors, on the transcript accumulation of the nine TdPDF genes, ten-day-old wheat seedlings were subjected to NaCl, PEG, MeJa, ABA, SA, or cold treatments and *F. garminearum* infection. Tissues were collected after 1, 3, 24, and 48 h. As illustrated in Figure 10A, the results of RT-qPCR showed that all these stresses induced the expression of most of the nine TdPDF genes. Salt stress enhanced the expression of TdPDF11 and TdPDF27 after 3 h and 24 h, respectively, of stress treatment. At this time of stress exposure, this induction reached the maximum, and it was about nine- and ten-fold, respectively, compared to the control condition. The highest increase in PEG-induced TdPDF expression was observed for TdPDF27, TdPDF17, and TdPDF25. Cold treatments significantly increased the expression level of TdPDF17 and TdPDF15 five- and six-fold, respectively, after 48 h. Under MeJa treatment, the expression levels of TdPDF11 and TdPDF15 increased approximately eight-and six-fold, respectively, after 48 h of stress application. The upregulation of TdPDF15 and TdPDF23 were observed after 48 h of ABA treatment, whereas it was only slightly induced by SA treatment.

In contrast, during the biotic stress treatment, all TdPDF genes were pronouncedly expressed in tissues inoculated by *F. graminearum* at 1, 3, 24, and 48 h (Figure 10B). These results confirm that the TdPDF genes may be associated with response to pathogen attack, consistent with the prediction of the PDF gene function. The results from this experiment demonstrated that analyzed TdPDF genes play distinctive roles in durum wheat responses to exogenous phytohormone treatments, which resemble phytohormonal cascades activated upon several abiotic or biotic stressors. Direct treatments with abiotic and biotic stress factors confirmed the involvement of TdPDF genes in plant reactions to a wide range of environmental stimuli.

## 4. Discussion

Defensins are proteins that may have either overlapping or distinct roles in plant defense and development. Although *PDF* genes have been identified in various plants, they have not been previously explored in durum wheat. Wheat defensins are particularly crucial due to wheat’s vulnerability to diseases like Fusarium head blight, which can severely impact yield and quality [60]. These defensins are distinct from those found in other crops, where the immune response to such pathogens is less pronounced [61,62]. Therefore, in this study, we identified 28 *TdPDF* genes in durum wheat using bioinformatics tools. This number is significantly lower than in common wheat (*n* = 73). However, the number of *PDF* genes varies considerably across species. This variation scan impacts their resistance to pathogens, stress adaptation, and overall resilience, contributing to evolutionary advantages and potential for crop improvement. One defensin (*Tm-AMP-D1*) was found in *T. monococcum* seeds, two in barley seeds (*γ-hordothionin* (*γ-H*) and *ω-hordothionin* (*ω-H*), six (*Sd1–6*) in sugarcane (*Saccharum* spp.), eight in *T. kiharae*, 11 in rice, and 15 in maize [17,29,63,64,65,66,67,68,69,70,71]. Model dicot *A. thaliana* possesses 15 *PDF* genes, while in legumes, the number usually ranges from several to over a dozen (for instance, from four in *Vigna angularis* to 22 in chickpea) [17,29,63,64,65,66,67,68,69,70,71]. Much higher numbers of sequences was found in the genomes of *Brassica napus*-37, and *Vitis vinifera*-79 [33,69].

In durum wheat, all 28 TdPDF proteins contain a gamma-thionin domain (PF00304), which is highly conserved across species and has been identified in PDF proteins from various plants [72]. Gene-family evolution is strongly influenced by tandem, segmental, and whole-genome duplications [73]. Our results indicate that both tandem and segmental duplications play a role in the evolution of *TdPDF* genes. Notably, many *TdPDF* genes located on the same chromosome are positioned near each other, suggesting that they likely originate from segmental duplication events and, therefore, may share similar functions or be involved in related biochemical pathways [55]. Duplications may also contribute to the evolution of distinct *PDF* paralogs with specialized roles in stress tolerance [74]. Such duplications could lead to functional differentiation, with some copies evolving to better defend against biotic stresses, such as fungal pathogens, while others may play a role in mitigating abiotic stresses like drought or salinity [75]. Recent studies have highlighted that gene duplication events allow for the accumulation of beneficial mutations, enhancing the adaptability of plants to changing environments [76]. Consistent with previous findings [56,57], our analysis confirmed that all TdPDF proteins contain a signal peptide. This signal peptide mediates the transport of defensins to the extracellular space, where they likely perform their main biological functions [58]. In silico analysis predicted extracellular localization for most TdPDF proteins, while one is localized in the mitochondrion. This variation in the localization of proteins in the cell may be related to factors such as protein-protein interaction. This result aligns with previous studies, which report extracellular localization for PDF proteins in rice [59], *Arabidopsis thaliana* [77], peanut [78], lentil [79], and *Brassica napus* [34]. Defensin genes are marked by a typical exon–intron–exon structure, with some exceptions previously reported [70,80]. All identified *TdPDF* genes contain a single intron with variable length, while the sizes of the two exons remain relatively conserved. These findings are consistent with other reports that indicated that most of the plant defensins feature a single intron [81,82]. Gene structure and physicochemical property analyses showed that *PDF* genes exhibit highly conserved architecture [83]. Molecular weight analysis classified durum wheat PDFs as a short peptide with a relatively low molecular weight. Their three-dimensional structure consists of one α-helix and three antiparallel β-sheets, stabilized by disulfide bonds. This is in agreement with previous studies [26,69,84]. The structural homologies observed in defensins highlight phylogenetic relationships between plant species, with PDFs serving as effective phylogenetic markers [20].

All *TdPDF* genes are implicated in the plant’s responses to a range of abiotic stresses, as revealed through promoter region analysis. Examining the promoter regions of genes is crucial for uncovering their potential functions [85]. The identification of GA-responsive cis-elements (such as P box, GARE motifs, and TATC box) suggests that these genes are likely regulated by GA and may play a role in GA signaling pathways and seed development. Similarly, the presence of drought-related cis-elements (DREs and MBS) indicates their potential involvement in drought resistance. Additionally, other cis-acting elements, including endosperm-related elements (AAGAA motifs and GCN4 motifs) and meristem-related motifs (CAT box and CCGTCC box), were found, implying that these genes may be important for plant development and tissue-specific expression. The abscisic acid-responsive cis-acting element ABRE and the core promoter elements TATA box and CAAT box in the promoter of the wheat *PDF* gene are all growth- and development-related elements. This suggests that *TdPDF* is associated with plant growth and development. A comparison of the cis-acting elements in the *PDF* genes of common and durum wheat revealed that some genes share similar elements in their promoter regions. The diversity of cis-regulatory elements in the promoter regions of *PDF* genes plays a fundamental role in the regulation of various pathways. In addition, identifying stress-responsive *TdPDF* genes offers significant potential for enhancing durum wheat productivity in climate-stressed regions. These genes are integral in plant defense mechanisms under abiotic stress conditions such as drought, heat, and salinity, which are exacerbated by climate change. Research has shown that *PDF* genes (chickpea defensin) help maintain plant stability by regulating stress-induced responses, reducing oxidative damage, and improving water-use efficiency [86]. Additionally, *PtDefensin* can increase disease resistance, as stressed plants are more susceptible to pathogens [87]. Understanding the molecular pathways governing these stress-responsive genes provides insights into how durum wheat can be adapted to thrive in harsher environments, enhancing yield stability. By integrating *PDF* genes into breeding programs, wheat varieties can be developed that are more resilient to climate change, reducing the need for external inputs such as irrigation and pesticides [88].

Plant defensins are generally recognized for their antimicrobial activity, primarily targeting fungi [89]. Defensins are known for their ability to bind and interact with phospholipids, key components of the fungal cell membrane, leading to pore formation and membrane disruption [29,90]. The primary mechanism of action for plant defensins is thought to involve the disruption of the pathogen’s cell membrane and wall integrity through the interactions between the positively charged regions of defensins and the negatively charged components of fungal membranes [91]. Additionally, defensins may bind to fungal wall components, inhibiting cell wall synthesis or enzyme activity, further impairing fungal growth [92].

PDFs disrupt microbial cell membranes by forming pores [93]. In contrast, chitinases target the cell walls of fungi by breaking down chitin and protease inhibitors block proteases, preventing pathogens from accessing plant proteins [94]. While thionins, another class of AMPs, are larger and more complex [95], TdPDFs are smaller and diffuse more rapidly through plant tissues, allowing them to quickly target pathogens and reduce pathogen load.

To verify the potential functions of *TdPDF* genes, we conducted an analysis of their expression profiles across various tissues and observed a wide range of expression patterns. Most *TdPDF* genes showed tissue-specific upregulation, including, specifically, *TdPDF13* in the leaf, *TdPDF15* in the root, *TdPDF25* in the embryo, *TdPDF27* in the anther, and *TdPDF25* and TdPDF23 in the seeds. In *Medicago sativa*, defensin genes are expressed in leaves, flowers, and seeds but not in roots, whereas in *Medicago truncatula*, their expression is limited to seeds and absent in other organs, corroborating that defensin expression is tissue-specific [82]. In *Populus trichocarpa*, the *PtDef* gene is expressed in petioles, roots, stems, and leaves, with the highest expression observed in petioles, followed by roots, stems, and leaves [96]. *ArabidopsisPDF2.2* is expressed in all organs of healthy plants except in the stems and seeds, and the expression of *PDF2.2* in the leaves can change upon infection by the pathogen *Alternaria brassicicola* [97]. Plant defensins have also been found in leaves, floral organs, tubers, fruit, and roots, and their expression in various plant organs provides a first line of defense against pathogen attack [19,56,83,84,98,99,100]. According to the literature, seeds are the most abundant source of defensins [19]. Seed defensins suppress fungal growth, enhancing seedling survival rate [101]. Similarly, in durum wheat, *TdPDFs* are substantially abundant in seedlings and storage tissues, suggesting their role in plant growth and development.

Abiotic stressors like heat, drought, salinity, and nutrient deficiencies are major contributors to global crop losses [102]. Recently, the role of antimicrobial peptides (AMPs) in plant responses to abiotic stress is becoming increasingly acknowledged [103], along with the involvement of certain plant defensins [23,86,104]. Nevertheless, our understanding of the function of defensin’s abiotic stress responses remains incomplete. In the present study, we found that most *TdPDF* genes were regulated by abiotic stress, as well as the stress-related phytohormones ABA, MeJa, and SA. The diverse expression patterns of various *TdPDF* genes in response to phytohormone treatments suggest their involvement in a range of physiological reactions to stress factors. Our findings are in line with those of previous studies on other species [17,29], where heat and drought stress could trigger the expression of chickpea defensins in *Arabidopsis thaliana* [86], and the *Dhn8* (dehydrin protein) from *Hordeum vulgare* L. was highly expressed in leaves under drought treatment [26]. In addition, the expression levels of *OsDEF7* and *OsDEF8* were affected by several stressful conditions [59]. Considering biotic stress response, *TdPDF* expression was upregulated during *F. graminearum* infection. Similarly, the expression levels of *TaPDFs* were differentially up-regulated by this pathogen [17]. Studies have demonstrated that *PsDef1* levels rise significantly in Scots pine seedlings during germination and in response to pathogenic infection by *Heterobasidion annosum* [105]. Also, when *Medicago truncatula* is infected by *Xanthomonas campestris*, the expressions of the *MtDef5* gene are upregulated, suggesting their antibacterial role [22]. Upregulation of *PDFs* under pathogen infection was also reported in pea, tobacco, *Arabidopsis*, and spruce [26]. Our results demonstrated that TdPDFs bind to phospholipids, suggesting that a similar mechanism may occur during contact with the fungal cell membrane. Such binding triggers pore formation and destabilizes membrane integrity, which may likely contribute to the inhibition of fungal growth, as suggested by Thevissen et al. [106] and observed in previous studies [29,90]. Additionally, research by Shahzad et al. [104] on representatives of *Arabidposis* genes revealed that apart from being involved in zinc tolerance, *AhPDF1s* in *A. halerii* exhibits antifungal properties. To validate the bioinformatics predictions made in the study, future studies should incorporate experimental approaches such as gene editing (CRISPR-Cas9) for knockout or overexpression studies in durum wheat, heterologous expression in *Arabidopsis* or *E. coli*, and integration with omics technologies like proteomics and metabolomics to validate gene function. Additionally, gene co-expression analysis and phenotypic assessment can further confirm the predicted roles of these genes.

## 5. Conclusions

In conclusion, this study provides the first comprehensive genome-wide analysis of the defensin gene family in durum wheat. A total of 28 *TdPDF* genes were identified and analyzed in terms of their structure, localization, phylogenetic relationships, conserved domain organization, protein interaction, docking analysis, and tissue-specific expression patterns. It allowed for comprehensive characterization of this gene family in durum wheat, as well as for gaining an insight into the structure, functions, and regulation of their encoded proteins. Due to a thorough examination of nine *TdPDF* gene expression profiles under abiotic and biotic stress conditions, novel information was gathered concerning the role of defensins in durum wheat’s response to environmental stimuli. These findings could serve as valuable resources for improving stress tolerance in wheat and other crop plants.

## Figures and Tables

**Table 1:** Overview of chromosomal position and characteristics of *TdPDF* genes in durum wheat.

Gene Name	Gene ID	Chr	Start Site	End Site	Length (bp)	CDS	Exon Number
*TdPDF1*	TRITD1Av1G013900.1	1A	29,234,920	29,235,726	352	249	2
*TdPDF2*	TRITD1Av1G003410.1	1A	7,557,945	7,558,278	334	249	2
*TdPDF3*	TRITD1Av1G013870.1	1A	29,069,143	29,069,486	344	249	2
*TdPDF4*	TRITD1Av1G013880.1	1A	29,172,467	29,172,850	384	297	2
*TdPDF5*	TRITD1Av1G013910.1	1A	29,237,984	29,238,315	332	249	2
*TdPDF6*	TRITD1Bv1G017710.1	1B	45,983,093	45,983,444	352	249	2
*TdPDF7*	TRITD1Bv1G003150.1	1B	7,441,922	7,442,263	342	252	2
*TdPDF8*	TRITD1Bv1G003190.1	1B	7,501,776	7,502,128	353	246	2
*TdPDF9*	TRITD1Bv1G017720.1	1B	46,017,987	46,018,314	328	225	2
*TdPDF10*	TRITD2Av1G011110.1	2A	19,957,063	19,957,436	374	228	2
*TdPDF11*	TRITD2Av1G011190.1	2A	20,339,771	20,340,134	364	234	2
*TdPDF12*	TRITD2Bv1G014190.1	2B	29,124,967	29,125,340	374	228	2
*TdPDF13*	TRITD2Bv1G014230.1	2B	29,274,981	29,275,371	391	237	2
*TdPDF14*	TRITD2Bv1G187060.1	2B	553,467,008	553,467,369	362	258	2
*TdPDF15*	TRITD3Av1G250280.1	3A	675,334,189	675,334,511	323	231	2
*TdPDF16*	TRITD3Av1G250300.1	3A	675,335,903	675,336,223	321	225	2
*TdPDF17*	TRITD3Av1G250390.1	3A	675,594,401	675,594,894	494	372	2
*TdPDF18*	TRITD3Bv1G242100.1	3B	736,591,016	736,591,326	311	219	2
*TdPDF19*	TRITD4Bv1G184330.1	4B	618,745,628	618,745,970	343	249	2
*TdPDF20*	TRITD4Bv1G184350.1	4B	618,748,241	618,748,486	246	246	1
*TdPDF21*	TRITD4Bv1G184360.1	4B	618,758,106	618,758,354	249	249	1
*TdPDF22*	TRITD5Av1G239070.1	5A	622,552,360	622,552,608	249	249	1
*TdPDF23*	TRITD5Bv1G174690.1	5B	511,145,927	511,146,428	502	405	2
*TdPDF24*	TRITD6Av1G019320.1	6A	45,820,912	45,821,245	334	249	2
*TdPDF25*	TRITD6Bv1G028170.1	6B	77,285,794	77,286,146	353	249	2
*TdPDF26*	TRITD6Bv1G028180.1	6B	77,294,347	77,294,682	336	249	2
*TdPDF27*	TRITD6Bv1G069150.1	6B	202,420,199	202,420,599	401	261	2
*TdPDF28*	TRITD7Bv1G138710.1	7B	436,196,050	436,196,380	331	252	2

CDS: coding sequence; Chr: chromosome.

**Table 2:** Physiochemical characteristics and predicted subcellular localization of TdPDF proteins.

Gene Name	Gene ID	Protein Length	Pl	MW (KDa)	Ii	GRAVY	Subcellular Localization	Signal Peptide Position
*TdPDF1*	TRITD1Av1G013900.1	82	8.92	8.945	58.10	0.029	Extracellular	1–33
*TdPDF2*	TRITD1Av1G003410.1	82	8.90	8.972	46.25	−0.017	Extracellular	1–33
*TdPDF3*	TRITD1Av1G013870.1	82	8.50	8.823	66.51	0.054	Extracellular	1–33
*TdPDF4*	TRITD1Av1G013880.1	98	9.79	10.612	72.63	0.265	Extracellular	1–33
*TdPDF5*	TRITD1Av1G013910.1	82	8.92	8.837	60.72	0.178	Extracellular	1–33
*TdPDF6*	TRITD1Bv1G017710.1	82	8.72	8.973	55.86	−0.060	Extracellular	1–33
*TdPDF7*	TRITD1Bv1G003150.1	83	9.10	9.080	51.22	−0.060	Extracellular	1–34
*TdPDF8*	TRITD1Bv1G003190.1	81	8.50	8.977	58.07	−0.265	Extracellular	1–32
*TdPDF9*	TRITD1Bv1G017720.1	74	8.17	7.942	42.43	0.177	Extracellular	1–18
*TdPDF10*	TRITD2Av1G011110.1	75	5.72	7.949	64.70	0.313	Extracellular	1–28
*TdPDF11*	TRITD2Av1G011190.1	77	8.47	8.295	46.32	0.386	Extracellular	1–27
*TdPDF12*	TRITD2Bv1G014190.1	75	5.72	7.947	70.96	0.299	Extracellular	1–28
*TdPDF13*	TRITD2Bv1G014230.1	78	8.48	8.359	42.82	0.373	Extracellular	1–28
*TdPDF14*	TRITD2Bv1G187060.1	85	8.91	9.857	33.90	−0.455	Extracellular	1–32
*TdPDF15*	TRITD3Av1G250280.1	76	8.14	8.424	20.38	0.242	Extracellular	1–20
*TdPDF16*	TRITD3Av1G250300.1	74	8.46	8.132	38.15	0.264	Extracellular	1–29
*TdPDF17*	TRITD3Av1G250390.1	123	6.63	13.426	51.05	−0.145	Mitochondrion	-
*TdPDF18*	TRITD3Bv1G242100.1	72	8.47	8.003	22.45	0.215	Extracellular	1–25
*TdPDF19*	TRITD4Bv1G184330.1	82	9.60	9.076	63.91	−0.160	Extracellular	1–33
*TdPDF20*	TRITD4Bv1G184350.1	81	9.54	8.644	42.15	0.086	Extracellular	1–33
*TdPDF21*	TRITD4Bv1G184360.1	82	9.49	8.912	26.87	−0.144	Extracellular	1–33
*TdPDF22*	TRITD5Av1G239070.1	82	9.69	8.945	26.52	−0.133	Extracellular	1–33
*TdPDF23*	TRITD5Bv1G174690.1	80	6.50	8.666	34.92	0.216	Extracellular	-
*TdPDF24*	TRITD6Av1G019320.1	82	9.22	8.761	43.00	0.195	Extracellular	1–26
*TdPDF25*	TRITD6Bv1G028170.1	82	9.22	8.819	40.65	0.155	Extracellular	1–33
*TdPDF26*	TRITD6Bv1G028180.1	82	9.08	8.885	58.38	0.113	Extracellular	1–33
*TdPDF27*	TRITD6Bv1G069150.1	86	5.55	9.498	61.52	−0.117	Extracellular	1–21
*TdPDF28*	TRITD7Bv1G138710.1	83	8.16	9.170	37.04	−0.212	Extracellular	1–34

PI: isoelectric point, MW: molecular weight (KDa), Ii: instability index, GRAVY: grand average of hydropathicity; -: not identified.

**Table 3:** TdPDF gene duplication and their orthologs in *T. aestivum*.

*T. durum*					*T. aestivum*	
Exon Number	Gene	Duplicate Gene	Homologs/ Paralogs	Duplication Type	Orthologs	Exon Number
2	*TdPDF3*	*TdPDF4*	Paralogs	Segmental duplication	*TaPDF1*	2
2	*TdPDF1*	-	-	-	-	-
2	*TdPDF7*	*TdPDF2*	Homologs	Whole-genome duplication	*TaPDF2*	2
2	*TdPDF7*	*TdPDF28*	Paralogs	Segmental duplication	*TaPDF2*	2
2	*TdPDF8*	*TdPDF28*	Paralogs	Segmental duplication	*TaPDF3*	2
2	*TdPDF6*	*TdPDF3*	Homologs	Whole-genome duplication	*TaPDF1*	2
2	*TdPDF6*	*TdPDF9*	Paralogs	Segmental duplication	*TaPDF1*	2
2	*TdPDF6*	*TdPDF4*	Homologs	Whole-genome duplication	*TaPDF1*	2
2	*TdPDF12*	*TdPDF10*	Homologs	Whole-genome duplication	*TaPDF5*	2
2	*TdPDF13*	*TdPDF11*	Homologs	Whole-genome duplication	*TaPDF6*	2
2	*TdPDF13*	*TdPDF12*	Paralogs	Segmental duplication	*TaPDF6*	2
2	*TdPDF15*	*TdPDF16*	Paralogs	Segmental duplication	*TaPDF10*	3
2	*TdPDF17*	*TdPDF16*	Paralogs	Segmental duplication	*TaPDF13*	2
2	*TdPDF18*	*TdPDF15*	Homologs	Whole-genome duplication	*TaPDF11*	2
1	*TdPDF20*	*TdPDF21*	Paralogs	Segmental duplication	*TaPDF15*	1
1	*TdPDF21*	*TdPDF19*	Paralogs	Segmental duplication	*TaPDF14*	4
1	*TdPDF22*	*TdPDF21*	Homologs	Whole-genome duplication	*TaPDF20*	1
2	*TdPDF24*	*TdPDF25*	Homologs	Whole-genome duplication	*TaPDF21*	2
2	*TdPDF24*	*TdPDF5*	Paralogs	Segmental duplication	*TaPDF21*	2
2	*TdPDF26*	*TdPDF14*	Paralogs	Segmental duplication	*TaPDF25*	2
2	*TdPDF26*	*TdPDF11*	Paralogs	Segmental duplication	*TaPDF25*	2

-: Not identified.

**Table 4:** Ka/Ks analysis for the duplicated *TdPDF* genes in durum wheat.

Gene	Duplicate	Ka	Ks	Ka/Ks
*TdPDF 3*	*TdPDF4*	0.2146	0.3813	0.5629
*TdPDF7*	*TdPDF2*	0.0328	0.1481	0.2219
*TdPDF7*	*TdPDF28*	0.1199	0.0891	1.3454
*TdPDF8*	*TdPDF28*	0.0508	0.0736	0.6903
*TdPDF6*	*TdPDF3*	0.0551	0.1738	0.3173
*TdPDF6*	*TdPDF9*	0.2252	0.3708	0.6073
*TdPDF6*	*TdPDF4*	0.2422	0.5648	0.4287
*TdPDF12*	*TdPDF10*	0.0298	0.0577	0.5167
*TdPDF13*	*TdPDF11*	0.0172	0.1178	0.1462
*TdPDF13*	*TdPDF12*	0.2303	0.5163	0.4461
*TdPDF15*	*TdPDF16*	0.0333	0.0555	0.5998
*TdPDF17*	*TdPDF16*	0.2330	1.0568	0.2204
*TdPDF18*	*TdPDF15*	0.0243	0.1092	0.2229
*TdPDF20*	*TdPDF21*	0.1077	0.1375	0.7835
*TdPDF22*	*TdPDF21*	0.0109	0.0665	0.1653
*TdPDF24*	*TdPDF25*	0.0161	0.0531	0.3038
*TdPDF24*	*TdPDF5*	0.1132	0.1997	0.5669
*TdPDF26*	*TdPDF14*	0.3258	0.4370	0.7454

## Data Availability

All data are contained within the article or Supplementary Materials.

## Supplementary Materials

The following supporting information can be downloaded at: https://www.mdpi.com/article/10.3390/biology14040404/s1, Table S1: Sequences of primers used in RT-qPCR analysis.
