# Supplementary material for: Genome-Wide Identification of the Defensin Gene Family in Triticum durum and Assessment of Its Response to Environmental Stresses
Source: Biology (Basel). 2025 Apr 11;14(4):404. doi: 10.3390/biology14040404 (PMC12024934; doi:10.3390/biology14040404)
Supplement: Supplementary file 1 [file biology-14-00404-s001.zip › biology-3538813-supplementary.pdf]

**Table S1.** Sequences of primers used in RT-qPCR analysis.

| Primers | Sequences |
|---|---|
| *qTdPDF25-F* | 5′- CTCCTCCTCGCCACAGAGAT-3′ |
| *qTdPDF25-R* | 5′- GACCCTCTTGCAGAAGCACT-3′ |
| *qTdPDF9-F* | 5′-CTCCTCCTGCTCTTCCTCCT-3′ |
| *qTdPDF9-R* | 5′-AAGCACTTGCCCTCGAAGT-3′ |
| *qTdPDF23-F* | 5′-GCTCCTGCTTTCCTCATACG-3′ |
| *qTdPDF23-F* | 5′-AGCGCCTGAATTTGCAGTAG-3′ |
| *qTdPDF13-F* | 5′- CCTGCTGCTAGTTGTCACCA-3′ |
| *qTdPDF13-R* | 5′- GCAAATGCACTTCCTCTTCA-3′ |
| *qTdPDF11-F* | 5′-TGGCGTCATCACACAAGTTC-3′ |
| *qTdPDF11-R* | 5′-TAAGGCAAATGTTGGCACAG-3′ |
| *qTdPDF27-F* | 5′-TCTTACCTTGGGTGCAGAGG-3′ |
| *qTdPDF27-R* | 3′-CAGCTCGCCACACTTCTTAG-3′ |
| *qTdPDF17-F* | 5′- AAGGTCTTCGTGGTCCTCCT-3′ |
| *qTdPDF17-R* | 5′- TAGCAGTCCTTGGTGCAGTG-3′ |
| *qTdPDF20-F* | 5′- CTCCACGCAAGCTCGTCT-3′ |
| *qTdPDF20-R* | 5′- CTCGGTCCTGCAGACGTT-3′ |
| *qTdPDF15-F* | 5′-TCTTGTGACCGCAGAGGAC-3′ |
| *qTdPDF15-R* | 3′-CAGTAACAACGGTGCCAAAA-3′ |
| *qTdCDC-F* | 5′-GCCTGGTAGTCGCAGGAGGAT-3′ |
| *qTdCDC-R* | 5′-ATGTCTGGCCTGTTGGTAGC-3′ |
